# Human Hepatocyte Nuclear Factors (HNF1 and LXRb) Regulate CYP7A1 in HIV-Infected Black South African Women with Gallstone Disease: A Preliminary Study

**DOI:** 10.3390/life13020273

**Published:** 2023-01-18

**Authors:** Suman Mewa Kinoo, Pragalathan Naidoo, Bhugwan Singh, Anil Chuturgoon, Savania Nagiah

**Affiliations:** 1 Discipline of Medical Biochemistry, School of Laboratory Medicine and Medical Science, College of Health Science, University of KwaZulu Natal, Glenwood, Durban 4041, South Africa; 2 Discipline of General Surgery, School of Clinical Medicine, College of Health Science, University of KwaZulu Natal, Umbilo, Durban 4001, South Africa; 3 Department of Human Biology, Medical School, Faculty of Health Sciences, Nelson Mandela University, Missionvale, Port Elizabeth 6065, South Africa

**Keywords:** HIV, gallstones, HNF1, LXRb, CYP7A1

## Abstract

Female sex, high estrogen levels, aging, obesity, and dyslipidemia are some of the risk factors associated with gallstone formation. HIV-infected patients on combination antiretroviral therapy (cART) are more prone to hypercholesterolemia. Bile acid synthesis is initiated by cholesterol 7-alpha hydroxylase (CYP7A1) and regulated by hepatocyte nuclear factors (HNF1α, HNF4α, and LXRb). The aim of this study was to evaluate the expression of HNF1α, HNF4α, LXRb, and miRNAs (HNF4α specific: miR-194-5p and miR-122^*^_1) that regulate CYP7A1 transcription in HIV-infected Black South African women on cART and presenting with gallstones relative to HIV-negative patients with gallstone disease. Females (*n* = 96) presenting with gallstone disease were stratified based on HIV status. The gene expression of *CYP7A1*, *HNF1α*, *HNF4α*, *LXRb*, miR-194-5p, and miR-122^*^_1 was determined using RT-qPCR. Messenger RNA and miRNA levels were reported as fold change expressed as 2^−ΔΔCt^ (RQ min; RQ max). Fold changes >2 and <0.5 were considered significant. HIV-infected females were older in age (*p* = 0.0267) and displayed higher low-density lipoprotein cholesterol (LDL-c) (*p* = 0.0419), *CYP7A1* [2.078-fold (RQ min: 1.278; RQ max: 3.381)], *LXRb* [2.595-fold (RQ min: 2.001; RQ max: 3.000)], and *HNF1α* [3.428 (RQ min: 1.806; RQ max: 6.507] levels. *HNF4α* [0.642-fold (RQ min: 0.266; RQ max: 1.55)], miR-194-5p [0.527-fold (RQ min: 0.37; RQ max: 0.752)], and miR-122*_1 [0.595-fold (RQ min: 0.332; RQ max: 1.066)] levels were lower in HIV-infected females. In conclusion, HIV-infected women with gallstone disease displayed higher LDL-c levels and increased bile acid synthesis, which was evidenced by the elevated expression of CYP7A1, HNF1α, and LXRb. This could have been further influenced by cART and aging.

## 1. Introduction

The advent of combination antiretroviral therapy (cART) in human immunodeficiency virus (HIV) treatment was one of the most significant advances in modern medicine. The HIV epidemic went from a public health crisis to a chronic treatable disease as cART markedly extended the life expectancy of infected individuals. While the large scale rollout of cART has made a positive impact on the outcomes of HIV morbidity and mortality, there now emerges an unprecedented population of people aging with HIV [1,2,3]. This has led to a new phenomenon—an “epidemic within an epidemic”—with metabolic disorders becoming increasingly prevalent in HIV-positive individuals on cART. These range from acute to chronic adverse effects, with the most common being cardiovascular disease, lipodystrophy, diabetes, and metabolic syndrome [4,5,6,7,8]. Altered glucose metabolism and hypercholesterolemia are hallmarks of HIV patients on chronic cART [6,8,9]. Kato et al. (2020) reported higher low-density lipoprotein (LDL) levels in HIV-positive patients on antiretroviral therapy (ART) relative to ART-naïve patients [6]. Elevated circulating LDL cholesterol (LDL-c) contributes to several of the metabolic syndromes, including symptoms observed in people on cART. Despite the established prevalence of metabolic syndromes and altered cholesterol homeostasis in this population, there is a dearth of knowledge on gallstone disease, a disease closely linked to both these factors [10].

Cholesterol gallstones, the result of biliary cholesterol superseding its saturation point, causing cholesterol microcrystal formation [11], account for ~80% of all gallstones in Western populations [12]. The etiology of gallstone formation involves an interplay between genetic and environmental factors. Risk factors include obesity, female sex, high estrogen levels, aging, diabetes, and metabolic syndrome [13,14]. Historically, gallstone disease was the most prevalent in North America, South America, some European populations, and India [15]; however, the rapid rate of urbanization, high fat diets, and the influence of HIV on non-communicable diseases has produced a paradigm shift, warranting more investigation into developing countries and Sub-Saharan Africa [14]. Data collected in South Africa suggest a steady increase in gallstone disease in the Black African population over the past ten years [16,17]. Considering the HIV endemic setting, very little is known regarding HIV-positive patients on cART with gallstone disease [18].

Gallstones are the result of a shift in equilibrium of the triad of cholesterol, bile acid, and lecithin, favoring bile to exist in a lithogenic rather than a liquid state [11]. Bile acid synthesis is initiated by the rate-limiting enzyme cholesterol 7-alpha hydroxylase (CYP7A1) [19]. This enzyme is a member of a superfamily known as cytochrome P450 (CYP) monooxygenases which catalyze reactions in xenobiotic metabolism, steroid synthesis, and fatty acid metabolism. CYP7A1 catalyzes the conversion of cholesterol to 7-alpha-hydroxycholesterol, initiating bile synthesis. The CYP7A1 enzyme is regulated via multiple pathways, including a negative feedback loop from the hepato-enteric circulation of bile acids [20]. As CYP7A1 regulates the first step of bile acid synthesis, it has been extensively investigated in relation to gallstone formation. Several studies have evaluated genetic variations in the *CYP7A1* gene in relation to gallstone disease risk, with stronger links found to genetic predisposition in males compared to females [21,22,23,24], while a deficiency in the enzyme is associated with gallstone formation [25,26]. Considering that females are disproportionately affected by gallstone disease, and they make up the demographic at highest risk for new HIV infections in South Africa, alternative molecular mechanisms need to be investigated.

The transcription of the *CYP7A1* gene is regulated by transcription factors in response to fluctuations in bile acid and cholesterol. Hepatocyte nuclear factors (HNFs) are a group of transcription factors—predominantly expressed in the liver—that maintain metabolic homeostasis through the regulation of genes involved in glucose, cholesterol, and fatty acid metabolism [27]. Hepatocyte nuclear factor 1 alpha (HNF1α) and hepatocyte nuclear factor 4 alpha (HNF4α) are HNFs that respond to bile acid levels: HNF4α binds directly to *CYP7A1* [28], while HNF1α binds to CYP7A1 regulators—hepatic bile acid binding protein and Farnesoid X receptor (FXR) [29]. MicroRNAs (miRNAs), namely miR-194-5p and miR-122^*^_1, are chiefly regulated by HNF4α in the liver [30,31]. Liver X receptor α/β (LXR α/β) is closely related to FXRs and binds to the liver x receptor element (LXRE) of the LXR target genes. Among these target genes are regulators of reverse cholesterol transport, the most prominent being ATP-binding cassette (ABC-) G1, ABCG5, and ABCG8. The activity of these cellular efflux pumps determines cholesterol flux, thus regulating CYP7A1 activity in response to hepatic cholesterol concentration [32,33]. 

Nuclear factors such as HNFs and LXRs are key upstream regulators of hepatic cholesterol metabolism and bile acid synthesis. The dysregulation of these transcriptional regulators leads to pathogenic outcomes that underlie metabolic disorders. This present study sought to evaluate the expression of hepatic nuclear factors (HNF1α, HNF4α, and LXRb) and miRNAs (HNF4α specific: miR-194-5p and miR-122^*^_1) that regulate CYP7A1 transcription in HIV-positive Black South African women on cART and presenting with gallstones relative to HIV-negative patients with gallstone disease. 

## 2. Materials and Methods

### 2.1. Patient Recruitment

This study utilized a case-series design comparing the hepatic expression of cholesterol regulating genes in HIV-positive (case) and HIV-negative (control) patients presenting with symptomatic gallstones. Ethical approval was obtained from the University of KwaZulu Natal (UKZN) Biomedical Research Ethics Committee (BREC) (BE276/16). Patients undergoing cholecystectomy for gallstone disease (biliary cholic, cholecystitis, jaundice, and gallstone pancreatitis) at King Edward VIII Hospital, Durban, KwaZulu Natal, South Africa, from January–December 2017 were recruited. In total, 96 Black South African women provided informed consent (standard consent form in two official main languages, i.e., English and isiZulu) for the retrieval of a liver biopsy and the recording of patient clinical parameters, including age, race, BMI, family history of gallstones, and comorbidities (HIV, hypertension, diabetes, hypercholesterolemia). The study was carried out in accordance with the institutional guidelines.

Following the analysis of clinical parameters in all subjects, five HIV-negative (control) and five HIV-positive (cases) were selected for mRNA analysis to identify if hepatic nuclear factors were differentially regulated. All patients selected were of Black African ethnicity and female gender, with no co-morbidities (diabetes and hypertensive statin therapy, hepatitis infection, or tuberculosis treatment). All HIV-positive patients were on fixed dose combination (FDC) therapy, with CD4 counts above 500 cells/mm^3^ and undetectable viral loads. The FDC regimen consisted of 3 drugs, namely two nucleoside reverse transcriptase inhibitors (NRTIs) drugs [tenofovir disoproxil fumarate (TDF) and emtricitabine (FTC)] and one non-nucleoside reverse transcriptase inhibitor (NNRTI) [efavirenz (EFV)]. Common second-line agents used were protease inhibitors (PI) [lopinivar/ritonavir (Aluvia) or atazanavir/ritonavir]. None of the patients were on integrase strand transfer inhibitor (InSTI) [dolutegravir (DTG)] based therapies. 

### 2.2. RNA Extraction and Real Time Quantitative PCR (RT-qPCR)

Liver tissue (1 cm × 1 cm) was submerged in RNAlater^®^ Stabilization Reagent (Qiagen, Hilden, Germany) in 2 mL cryovials at collection and stored at −80 °C until RNA isolation. RNA was extracted using a chloroform-based method using Qiazol lysis buffer (Qiagen), per the manufacturer’s instructions. The purity and concentration of the crude RNA was assessed using the Nanodrop 2000 spectrophotometer (Thermo Fisher Scientific, Waltham, MA, USA). The crude RNA was standardized to a concentration of 1000 ng/μL and stored at −80 °C until further use.

Thereafter, 1 µg of the standardized RNA sample was used for complementary DNA (cDNA) synthesis using PCR. The miScript RT II kit (Qiagen) (miRNA gene expression studies) and QuantiTect Reverse Transcription kit (Qiagen) (gene expression studies) were used to create cDNA, per the manufacturer’s instructions.

Messenger RNA quantification (*CYP7A1*, *HNF1α*, *HNF4α,* and *LXRb*) and miRNA quantification (miR-194-5p and miR-122^*^_1) were performed using the Applied Biosystems Viia7 Real-Time PCR System (Thermo Fisher Scientific). 

MiRNA expression studies were performed using the miScript^TM^ SYBR^TM^ Green PCR kit (Qiagen, Hilden, Germany) and specific miScript Primer Assays for the above-mentioned miRNAs (Qiagen), according to the manufacturer’s instructions. Human RNA, U6 small nuclear 2 (*RNU6-2*), was used as a housekeeping gene. 

Messenger RNA gene expression studies were performed using the PowerUp™ SYBR™ Green Master Mix (Thermo Fisher Scientific) and specifically designed primer sequences for the above-mentioned genes (Inqaba Biotec, Ibadan, Nigeria), according to the manufacturer’s instructions. The sense and antisense primer sequences for the above-mentioned genes are shown in Table 1, along with their annealing temperatures. Housekeeping genes *GAPDH* and *18SrRNA* were concurrently quantified for normalization of the results. 

### 2.3. Statistical Analysis

Comparisons of clinical parameters between HIV-negative (control) and HIV-positive (cases) patients presenting with gallstones were conducted by performing a Mann–Whitney U Test. All data were analyzed using the GraphPad Prism 7 statistical software package. A result of *p <* 0.05 was considered statistically significant.

RT-qPCR analysis of mRNA levels was performed using the QuantStudio 7 Pro Real-Time PCR Systems Software (Thermo Fisher Scientific). Messenger RNA and miRNA levels were reported as relative fold change, expressed as 2^−ΔΔCt^ (RQ min; RQ max). Fold changes relative to the HIV-negative controls of >2 and <0.5 were considered significantly different, based on the calculation described above.

## 3. Results

### 3.1. Clinical Characteristics of Patients

The analysis of the clinical parameters is summarized in Table 2. The HIV-positive group was significantly older than the HIV-negative controls (*p* = 0.0267). The results show that the HIV-positive group had a lower BMI, with overall higher levels of total cholesterol, triglycerides, high density lipoprotein cholesterol (HDL-c), and significantly higher low-density lipoprotein cholesterol (LDL-c) (*p* = 0.0419).

### 3.2. Hepatic CYP7A1, LXRb, HNF1α, and HNF4α Gene Expression

**Figure 1:**
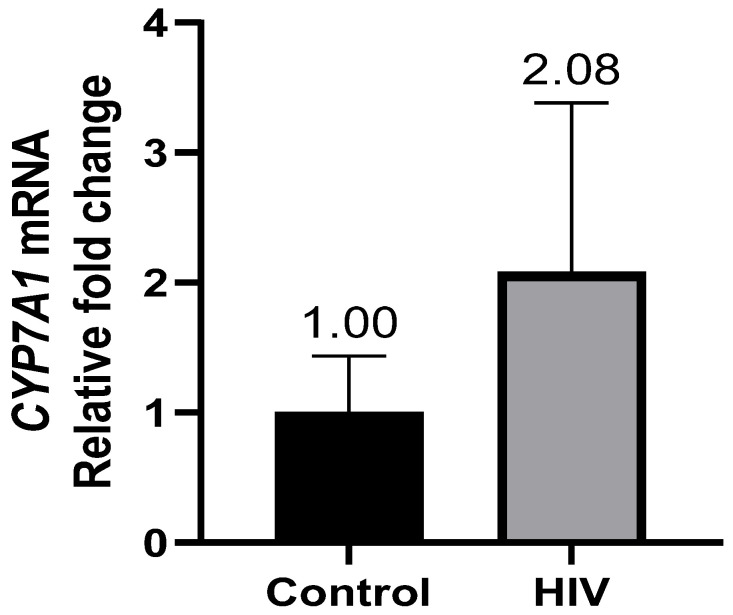
Hepatic *CYP7A1* transcript levels were significantly higher (>2-fold) in HIV-positive women relative to the control group.

**Figure 2:**
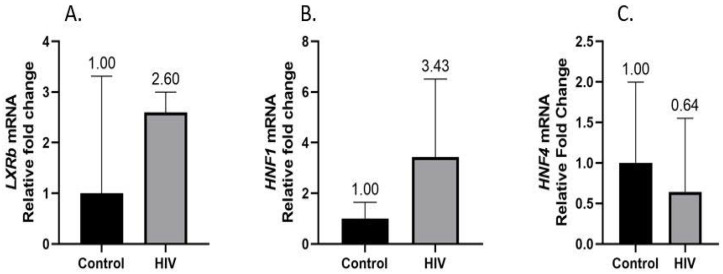
CYP7A1 regulators *LXRb* (**A**) and *HNF1α* (**B**) mRNA levels were significantly higher in HIV-positive patients with gallstone disease (>2-fold) relative to the control group. Hepatic *HNF4α* mRNA levels were lower in HIV-infected women (**C**).

RT-qPCR analysis showed that the hepatic mRNA levels of *CYP7A1* were significantly higher in women with HIV and gallstone disease relative to women with gallstone disease alone [2.078-fold (RQ min: 1.278; RQ max: 3.381)] (Figure 1). The transcriptional regulators of *CYP7A1* were subsequently quantified: *LXRb* [2.595-fold (RQ min: 2.001; RQ max: 3.000)] (Figure 2A) and *HNF1a* [3.428-fold (RQ min: 1.806; RQ max: 6.507] (Figure 2B) mRNA levels were concomitantly higher in the HIV-positive women compared to the HIV-negative control group. Hepatic *HNF4a* mRNA levels were lower in HIV-positive women [0.642-fold (RQ min: 0.266; RQ max: 1.55)] (Figure 2C). 

### 3.3. Hepatic miR-194-5p and miR-122^*^_1 Gene Expression

**Figure 3:**
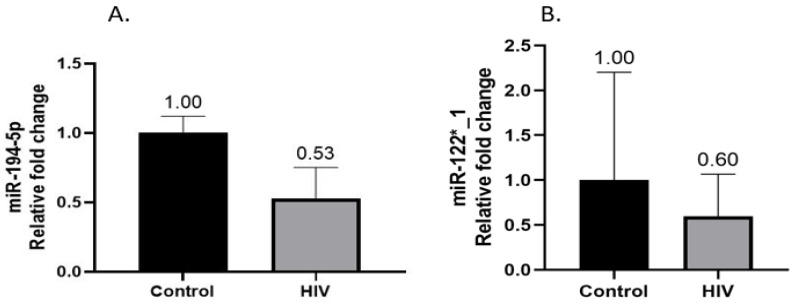
Hepatic expression of both miR-194-5p (**A**) and miR-122*_1 (**B**) was lower in HIV-positive patients with gallstone disease relative to the HIV-negative control group.

Hepatic miRNAs regulated by HNF4α were quantified by RT-qPCR. Both miR-194-5p [0.527-fold (RQ min: 0.37; RQ max: 0.752)] (Figure 3A) and miR-122*_1 [0.595-fold (RQ min: 0.332; RQ max: 1.066)] (Figure 3B) were observed at lower concentrations in HIV-positive women compared to HIV-negative women with gallstone disease.

## 4. Discussion

Gallstone disease is triggered by several risk factors, including female sex, aging, obesity, high estrogen levels, and dyslipidemia [13,14]. Within the context of an African population, age, waist circumference, and LDL-c were risk factors for gallstone disease in Sudanese individuals [34]. In Black South African women, BMI and continuous exposure to a Western lifestyle (characterized by high fat intake and low dietary fiber intake) were the main risk factors for gallstone disease [16]. 

Long-term use of cART has been linked to a number of metabolic diseases; however, there is a paucity of data on its association with gallstone disease in black South Africans [9,10,11,12]. It has been demonstrated in other populations that the accumulative exposure to atazanavir/ritonavir for over 2 years is associated with a 6.29-fold increase in the risk for incident cholelithiasis [18,35,36,37], while other studies report an increased rate of cholelithiasis (9.8%) in HIV-positive patients on protease inhibitor (PI)-inclusive cART [38]. HIV-infected patients on cART are more susceptible to hypercholesterolemia [6,9]. Despite newer integrase inhibitor (II) antiretroviral drugs showing lower lipid abnormalities than previously used PI-based ART, abnormalities in lipid concentration still occur, and this may be reflective either of the viral effects itself, chronic ART use, or persistent immune activation in HIV infection [39]. 

Atazanavir, ritonavir, and indinavir significantly decreased *CYP7A1* mRNA levels in rodent hepatocytes, indicating an effect of cART on the bile acid synthesis pathway [40,41]. A substantial amount of evidence dating back as far as 1975 [42], along with more recent evidence, has demonstrated that a deficiency in CYP7A1 [43], genetic variations in the CYP7A1 gene [44], and the inhibition of CYP7A1 with lipid lowering drugs such as fibrates [45], result in increased cholesterol excretion in bile, which increases the risk of gallstone formation [22,26]. Relative to Admirand’s theory, lower CYP7A1 activity compromises the conversion of cholesterol to bile acid, resulting in increased cholesterol excretion and decreased bile acid excretion, favoring gallstone formation [11]. In Chilean Hispanic and Mapuche subjects (known to have one of the highest incidences of gallstones worldwide), an increase in *CYP7A1* expression [46] and an increase in bile acid excretion [47] resulted in a higher incidence of gallstone formation. Our study showed that HIV-positive patients on cART displayed higher hepatic *CYP7A1* mRNA (Figure 1), suggesting a possible response to the impaired enterohepatic circulation of bile acids in HIV-positive patients [48]. It is worth noting that the studies demonstrating a cART-mediated reduction in *CYP7A1* mRNA levels have been conducted in PI-type antiretroviral drugs [40,41], while the patients in this study are on NRTI/NNRTI-based therapies. Further, the chronic inflammatory impact of HIV infection in conjunction with cART use must be considered as a factor influencing CYP7A1 expression. The accelerated aging phenotype observed in HIV-positive populations is a likely contributor to increased *CYP7A1* mRNA levels [49,50,51]. This finding warranted investigation into the transcriptional regulators of CYP7A1, namely HNF1α, HNF4α, and LXRb. 

The regulation of CYP7A1 by HNF1α has been studied extensively in mice, and it was found to downregulate CYP7A1 indirectly by binding to FXR receptors [29,52]. In a murine study, HNF1α was found to be a transcriptional regulator of FXR, and when HNF1α was inhibited, it resulted in the downregulation of FXR, with resultant gallstone formation [53]. Moschetta et al. (2004) reported that FXR knockout mice that were fed a lithogenic diet displayed an increased susceptibility to developing gallstones. The administration of the FXR agonist to the gallstone-susceptible mice decreased their susceptibility to gallstone formation [54]. Similar findings were reported elsewhere [55]. However, in a study by Chen et al. (1999), it was demonstrated that unlike in rodents, where HNF1α regulates CYP7A1 by binding to FXR, in humans HNF1α binds directly to CYP7A1 and increases its transcriptional activity, thus having the opposite effect to that observed in rodents [56]. Similar to the findings from Chen et al. (1999), in our study, HIV-infected patients with gallstones displayed increased expression of *CYP7A1* and *HNF1α* compared to their HIV-negative counterparts with gallstones, indicating that HIV may have an effect on HNF1α and CYP7A1. 

Another hepatic nuclear factor, HNF4α, binds directly to CYP7A1. Bile acids cause a negative feedback loop, resulting in the downregulation of CYP7A1, not only via HNF1α and FXR, but also HNF4α, which has a direct binding site on CYP7A1. Normally, HNF4α binds and upregulates CYP7A1; however, there is evidence that bile acids can downregulate CYP7A1 transcription by reducing the transactivation potential of HNF4α [28]. In our findings, however, among HIV-positive patients with gallstones, *HNF4α* was reduced in comparison to the levels in HIV-negative patients, yet *CYP7A1* was upregulated. This demonstrates that HNF4α on its own is not a regulator of CYP7A1 in the pathogenesis of gallstones in HIV-positive patients. This mirrors the results of other studies that demonstrated that HNF4α, together with LRH-1, COUP, TFII, and PCG-1α, is required for the upregulation of CYP7A1. HNF4α on its own is not capable of this [57,58]. Bacterial endotoxins, proinflammatory cytokines [59], TNF-α, and interleukin-1 produced by macrophages in the liver [60] are known to decrease the activity of CYP7A1. De Fabiani et al. (2001) demonstrated that HNF4α is the target transcriptional factor mediating the effects of these proinflammatory cytokines on CYP7A1 [28]. HIV-positive patients undergo a phenomenon of persistent inflammatory response, which may account for the decrease in *HNF4α* levels [61]. It must be noted that the HIV-positive group was older than the uninfected control group, with lower BMIs (Table 2). Aging is associated with the decreased expression of HNF4 and consequently, lower bile synthesis through the downregulation of CYP7A1. Bertolotti et al. (2008) demonstrated these findings in liver biopsies [62]. While BMI has not directly been linked to hepatic nuclear factors, a high fat diet has been shown to upregulate HNFs. Our previous paper on cholesterol signaling showed that despite having a lower BMI, this group displayed mRNA profiles similar to those expected in people with higher BMIs [63].

HNF4α is a key regulator of hepatic microRNAs miR-122 and miR-194. MiR-122 is found in abundance in the liver and plays an important role in regulating metabolic pathways, including fatty acid synthesis and cholesterol biosynthesis [31]. MiR-194 play a key role in hepatic cell functions [30]. In our study, *HNF4α* expression was lower in the HIV-infected group, which correlated with the decreased expression of miR-194-5p and miR-122^*^_1. This suggests that HNF4α may have more of an impact on microRNA expression in the liver than the CYP7A1-mediated bile acid synthesis pathway.

LXR agonists have been shown to bind to LXRE on mouse *CYP7A1,* causing its upregulation [64]. LXRE binds both LXRa and LXRb; however, LXRa binds more strongly than LXRb; thus, LXRa knockout mice exhibit more cholesterol accumulation. However, human CYP7A1 does not contain LXRE, and is thus shown not to be upregulated by LXRa agonists and seems to be more affected by diet-induced hypercholesterolemia due to its inability to convert cholesterol into bile salts [65]. Goodwin et al. (2003) demonstrated a downregulation of CYP7A1 by LXR in response to cholesterol loading [66]. Again, there is the implication that the regulation of the enterohepatic circulation may be a result of this downregulation as a compensation mechanism to decrease cholesterol absorption (by decreasing bile acid production) following a high cholesterol diet; however, in humans, this compensatory mechanism has been shown to be inefficient [67]. In our study, the findings are in keeping with those from mice studies in which an LXRb increase co-existed with an upregulation of CYP7A1, indicating that LXRb might well be a regulator of CYP7A1 and gallstone formation in these group of patients compared to that noted in HIV-negative patients with gallstones. Again this may be due to the underlying dysfunction of enterohepatic terminal ileum absorption in HIV-positive patients, causing LXRb to increase CYP7A1 in an attempt to produce more bile acid for absorption [68]. 

## 5. Conclusions

In summary, HIV-infected women with gallstone disease displayed higher LDL-c levels and increased bile acid synthesis, which was evident by the elevated expression of *CYP7A1*, *HNF1α,* and *LXRb*. This could have been further influenced by cART and aging. HNF4α, which is known to cause the upregulation of CYP7A1, was suppressed with the upregulation of CYP7A1 and LXR, known to cause the downregulation of CYP7A1 in humans as opposed to mice, having the opposite effect on HIV-infected women. 

The best theoretical explanation for this is an interruption in the enterohepatic circulation, as evidenced by HIV-positive patients known to have chronic inflammatory and relative malabsorptive disorders of the ileum, which may result in the upregulation of CYP7A1 to produce more bile salts. This is the most probable explanation for gallstone formation in HIV-positive patients; however, this hypothesis requires validation with further trials and a larger sample size. A limitation of this study is the small sample size used, as well as the fact that the age in the HIV-positive group was significantly higher than that in the control group. However, these preliminary findings can be the foundation for future studies that expand on sample size and compensate for the age discrepancy. Further, proof of concept can be performed by the inhibition of the investigated genes in in vitro or animal models to gain mechanistic insights into the individual players in the pathogenesis of gallstone disease in HIV infection. In conclusion, whole genome sequencing studies can provide a better understanding/insight into gallstone disease in Black African women.

## Figures and Tables

**Table 1:** Primer sequences and annealing temperatures for RT-qPCR.

Gene	Primer Sequence	Annealing Temperature (°C)
*HNF1α* sense *HNF1α* antisense	5′-ACCAAGCCGGTCTTCCATACT-3′ 5′-GGTGTGTCATAGTCGTCGCC-3′	58
*HNF4α* sense *HNF4α* antisense	5′-CACGGGCAAACACTACGGT-3′ 5′-TTGACCTTCGAGTGCTGATCC-3′	55
*LXRb* sense *LXRb* antisense	5′-AGAAGATTCGGAAACAACAGCA-3′ 5′-GCTGGATCATTAGTTCTTGAGCC-3′	53
*CYP7A1* sense *CYP7A1* antisense	5′-GAGAAGGCAAACGGGTGAAC-3′ 5′-GGATTGGCACCAAATTGCAGA-3′	54

**Table 2:** Clinical parameters of patients presenting with gallstones, stratified based on HIV status.

Parameters	HIV-ve	HIV+ve	*p*-Value
Age (years)	29.60 ± 5.41	39.60 ± 6.189	0.0267 *
BMI (kg/m^2^)	34.06 ± 5.980	32.63 ± 10.84	0.807
Total Cholesterol (mmol/L)	3.640 ± 1.107	4.882 ± 0.9883	0.0986
Triglycerides (mmol/L)	0.7640 ± 0.3886	0.8980 ± 0.4909	0.6457
HDL-c (mmol/L)	1.192 ± 0.2821	1.328 ± 0.5758	0.6526
LDL-c (mmol/L)	2.096 ± 0.7410	3.160 ± 0.6403	0.0419 *

* *p* < 0.05.

## Data Availability

The datasets used and/or analyzed during the current study are available from the corresponding authors on reasonable request.
